# Comparative transcriptomic analysis reveals genetic divergence and domestication genes in *Diospyros*

**DOI:** 10.1186/s12870-019-1839-2

**Published:** 2019-05-30

**Authors:** Changfei Guan, Shuyuan Liu, Mengke Wang, Hao Ji, Xiaofeng Ruan, Renzi Wang, Yong Yang

**Affiliations:** State Key Laboratory of Crop Stress Biology for Arid Areas, College of Horticulture, Northwest A & F University, Yangling, 712100 Shaanxi China

**Keywords:** *Diospyros*, Species, Comparative transcriptome, Genetic divergence, Domestication genes

## Abstract

**Background:**

Persimmon (*Diospyros kaki*) is the most economically cultivated species belonging to the genus *Diospyros*. However, little is known about the interspecific diversity and mechanism of domestication, partly due to the lack of genomic information that is available for closely related species of *D. kaki* (DK). Here, we performed transcriptome sequencing on nine samples, including DK, a variety of DK and seven closely related species, to evaluate the interspecific genetic divergence and to identify candidate genes involved in persimmon domestication.

**Results:**

We obtained a total of 483,421 unigenes with N50 at 1490 bp in the nine *Diospyros* samples and identified 2603 orthogroups that were shared among all the samples using OrthoMCL analysis. A phylogenetic tree was established based on the tandem 2603 one-to-one single copy gene alignments, showing that DK was closely related to *D. kaki* var. *silvestris* (DKV) and that it clustered with the clade of *D. deyangnsis* (DD) and was farthest from the *D. cathayensis* (DC) species. The nonsynonymous substitutions (Ka), via synonymous substitution (Ks) ratios, was directly proportional to the genetic relationship of the different species. The higher the Ka/Ks ratios, the longer the distance was. Moreover, 31 positively selected genes (PSGs) involved in carbohydrate metabolism and phenolic metabolism were identified and isolated, and nearly all PSGs except the *MATE* gene had a high expression in the DK or DKV species. It was hypothesized that these genes might contribute to the domestication of the DK species. Finally, we developed the expressed sequence tag-simple sequence repeat (EST-SSR) and identified 2 unique amplicons DKSSR10 and DKSSR39: the former was absent in the DC species but was present in the other species, the latter had a long amplification product in the DJ species.

**Conclusion:**

This study presents the first transcriptome resources for the closely related species of persimmon and reveals interspecific genetic divergence. It is speculated that DK is derived from the hybridization of DD and DO species. Furthermore, our analysis suggests candidate PSGs that may be crucial for the adaptation, domestication, and speciation of persimmon relatives and suggests that DKSSR10 and DKSSSR39 could potentially serve as species-specific molecular markers.

**Electronic supplementary material:**

The online version of this article (10.1186/s12870-019-1839-2) contains supplementary material, which is available to authorized users.

## Background

*Diospyros* Linn., which belongs to the Ebenaceae family, is the largest, most widely distributed and economically important genus, with over 500 species [1]. Asia and the Pacific region, in which approximately 300 species of the genus *Diospyros* are distributed, represent diversity centers [2, 3]. Persimmon (*D. kaki*, DK, 2n = 6x = 90; 2n = 9x = 135), which is believed to have originated in China, is the most economically important cultivated species of the genus *Diospyros*. Currently, the cultivation of the persimmon crop is rapidly expanding in Israel, Italy, and Spain in addition to the main cultivated countries of China, Japan, and Korea. *D. lotus* (DL, 2n = 2x = 30) is usually used as the root stock for DK, which is important for enlarging the cultivation range and improving fruit quality. Furthermore, there are some other species of *Diospyros* from ancient times that have a wide application. For instance, *D. oleifera* (DO, 2n = 2x = 30) is used as a wood paint due to its high content of tannin, and *D. jinzaoshi* (DJ, 2n = 2x = 30) and *D. cathayensis* (DC, 2n = 6x = 90) are cultivated for ornamental purposes. *D. virginiana* (DV, 2n = 6x = 90) and *D. glaucifolia* (DG, 2n = 2x = 30) are usually used as timber wood. However, there are few investigations about the interspecific genetic relationships in the genus *Diospyros* compared with the intraspecific phylogenetic relationships in the species DK. Previous studies showed that DK is closely related to DO-based chloroplast genome sequences and molecular markers, including inter-retrotransposon amplified polymorphisms (IRAPs) and retrotransposon microsatellite amplified polymorphisms (REMAPs) [4, 5]. Nevertheless, the analysis of sequence-specific amplified polymorphisms (SSAPs) and amplified fragment length polymorphisms (AFLPs) revealed that DK is more closely related to the DL species [4]. Moreover, some new species of the genus *Diospyros* have been discovered in recent years. Tang et al. reported that DJ might be a new species based on morphology as well as internal transcribed sequence (ITS) and *mat*K sequence analyses [6]. In our previous study, *D. deyangnsis* (DD, 2n = 4x = 60), which is distributed in Deyang city, Sichuan Province (China), was also considered a new special species according to chromosome number and SRAP markers [7]. Recently, Li et al. suggested that DK had a closer genetic relationship with DD than with DO [8]. Thus, with regard to the phylogenetic relationship between DK and other species in the genus *Diospyros*, further study is required.

The persimmon has at least 2600 years of cultivation history in China, according to the earliest written records in the ancient book of “Liji·Neize” [9]. The persimmon tree has been collected and domesticated since the BC 1000. With the development of grafting technology, large scale cultivation of persimmon appeared in the Tang and Song dynasties (AD 618–1279). It is noticeable that, the cultivation of the persimmon trees were further developed in the Ming and Qing dynasties (AD 1368–1911) since that persimmons were wildly considered as a valuable “woody gain” in that period [9]. Currently, the persimmon cultivated area in China reached 933,200 ha, accounting for 91.34% of the world’s cultivated area. Meanwhile, the cultivated areas in South Korea, Japan, and Spain accounted for 2.73, 2.08, and 1.30%, respectively (FAO, 2017).

As the most important and economically valuable species of the genus *Diospyros*, the quality and commercial value of DK is large, and it has a higher soluble sugar content and a lower tannin content in the fruit as a whole compared with other species. The molecular mechanism of domestication has been studied for over a century, but most previous studies have focused on a single or a few candidate genes in model systems [10, 11]. Based on reference genomes and next-generation sequencing (NGS) technologies, a total of 67 gene loci have been identified as potential targets for adaptation in the domestication of the apple [12]. In addition, over 50 positively selected genes in the tomato were detected based on sequence divergence using RNAseq technique [10]. However, due to complicated heredity traits and the limitation of the reference genome in the DK species, few investigations focusing on adaptation genes have been conducted.

Positively selected genes, with a higher divergence compared with the remaining pairs of genes in domesticated fruits, are assumed to be attributable to the power of artificial selection [12, 13]. The rapidly evolved genes are detected by evaluating the ratio of the nonsynonymous substitutions (Ka) and the synonymous substitutions (Ks) between orthologous coding regions [14, 15]. Paired genes with Ka/Ks > 1 are said to have experienced a rapid evolution by natural or artificial selection [16]. The genetic mechanism of domestication-associated phenotypes has been studied in several model plants, most notably in maize, rice, and the tomato [10, 17, 18]. The results demonstrated that the rapid phenotypic divergence associated with domestication is often due to some genetic loci [19]. The identification of rapidly evolving genes in non-model organisms is usually restricted by the availability and the cost of sequence information. The recent development of NGS provides an approach to acquire abundant and economical genomic data for non-model species, especially transcriptome sequences. RNA-sequencing is increasingly being used to study adaptive evolution and detect adaptive loci [10, 20]. Comparative transcriptome studies among closely related species have been conducted for various plants, such as the tomato [10], Berberidaceae [20], Primrose [21], and *Chrysanthemum* [22], which not only provide genus-specific SSR primers or single copy orthogroups but also detect some domestication-associated genes.

In the present study, we deep-sequenced the transcriptomes of nine samples to ascertain their genetic relationship and identify domestication-associated genes. The nine samples from the genus *Diospyros* included a domesticated persimmon (DK species), a variety of persimmon named *D. kaki* var. *silvestris* (DKV, also known as wild persimmon, 2n = 6x = 90), and seven wild species (DJ, DC, DV, DO, DG, DD, and DL). A comparative transcriptomic analysis of nine samples was carried out to identify orthogroups and to evaluate the genetic distance. Next, the ratio of the Ka and Ks for each ortholog pair was used to identify the positively selected genes among the nine samples and the domestication-associated genes. Finally, the genus-specific expressed sequence tag-simple sequence repeat (EST-SSR) markers from the putative single-copy genes were detected. Our analysis provides candidate genes for domestication in response to artificial selection in persimmon relatives and reveals some EST-SSRs that potentially serve as a species-specific molecular markers.

## Results

### Morphological characteristics and physiology evaluation

**Fig. 1:**
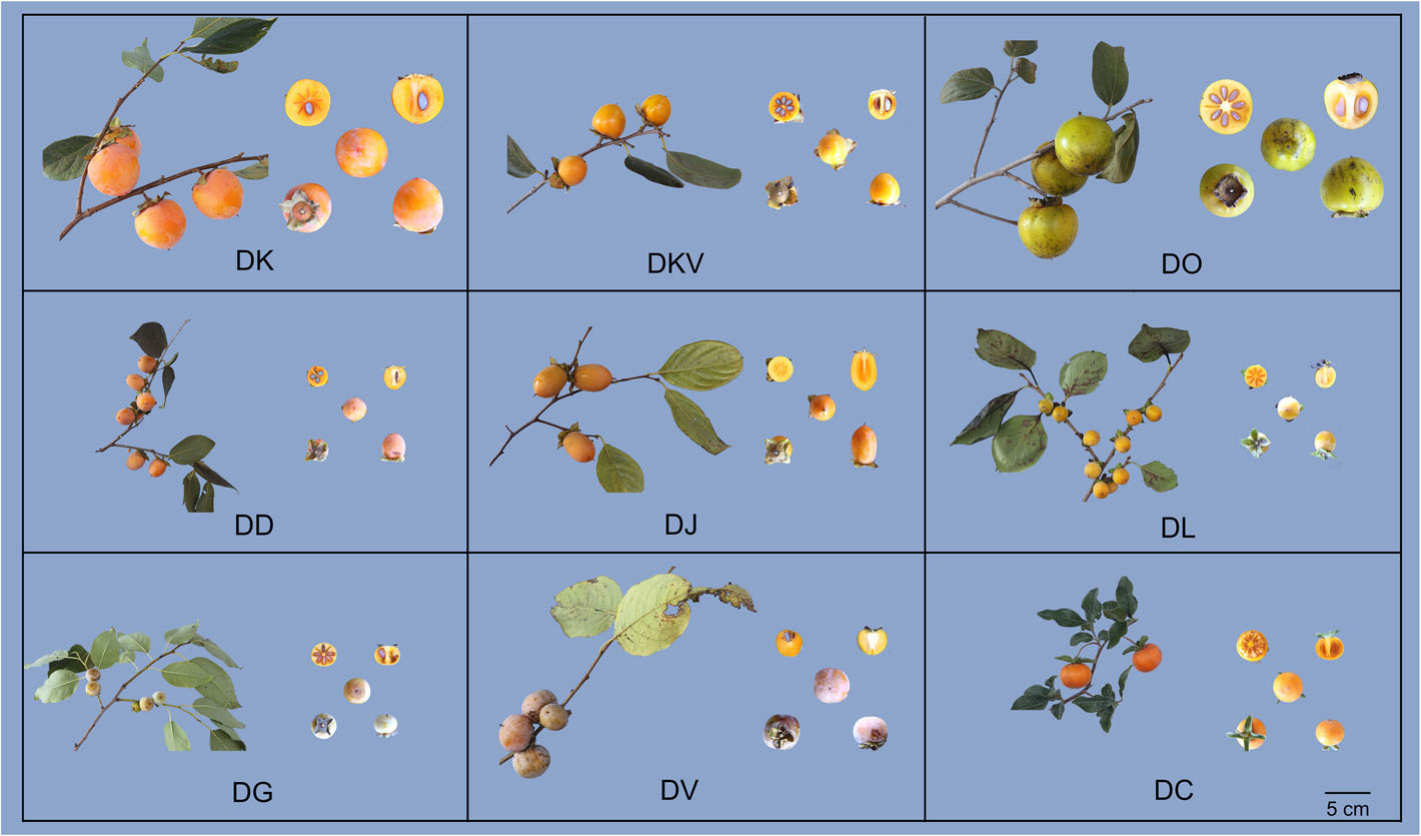
Morphological characteristics of the nine *Diospyros* samples. Photographs showing the leaves, stems, and fruits sampled on October 31, 2018 in the NFGP. *Bar* = 5 cm

The morphological characteristics including fruit, leaves and seed size present the significant differences among the nine samples (Fig. 1 and Table 1). DK and DO exhibited the larger fruit size compared with others samples. DK and DKV, as well as DL and DG had a similar leaf traits, respectively. DC had the thorn branches, the longest fruit stalk, the smallest fruits and leaf, showing the specific characters among the nine samples.

**Table 1:** The phenotypic characteristics of the nine *Diospyros* samples

Abbreviation	Accession name	Species	Chromosome ploidy	Origin	Colors of shoot	Leaf blade shape	Average fruit weight (g)	Fruit shape	Fruit skin color	Fruit development period (d)	Plant growth period (d)
DK	Huxian Huo Guan	*D. kaki*	2n = 6X = 90	Shaanxi, China	Brown	Long elliptic	78	Cordate	Orange-red	166	237
DKV	Yeshi	*D. kaki* var. *silvestris*	2n = 6X = 90	Zhejiang, China	Brown-grayness	Elliptic	23.2	Cordate	Orange-yellow	182	246
DO	Youshi	*D. oleifera*	2n = 2X = 30	Zhejiang, China	grayness	Wide elliptic	119.8	Cordate	Green	184	260
DD	Deyangshi	*D. deyangnsis*	2n = 4X = 60	Sichuan, China	Brown	elliptic	12	Ovate	Orange-red	156	236
DJ	Jinzaoshi	*D. jinzaoshi*	2n = 2X = 30	Zhejiang, China	Brown	elliptic	16	Long-cordate	Orange-yellow	178	253
DL	Junqianzi	*D. lotus*	2n = 2X = 30	Shaanxi, China	grayness	Wide elliptic	11.9	Ovate	Yellow-green	167	252
DG	Zhejiangshi	*D. glaucifolia*	2n = 2X = 30	Zhejiang, China	grayness	Lanceolate	12	Oblate	Light-yellow	165	249
DV	American Persimmon	*D. virginiana*	2n = 6X = 90	Beit Shemen, Israel	Brown-grayness	Elliptic	16.6	Oblate	Light-yellow	157	238
DC	Wushi	*D. cathayensis*	2n = 6X = 90	Henan, China	Dark-red	Lanceolate	11	Globe	Orange-red	172	258

**Fig. 2:**
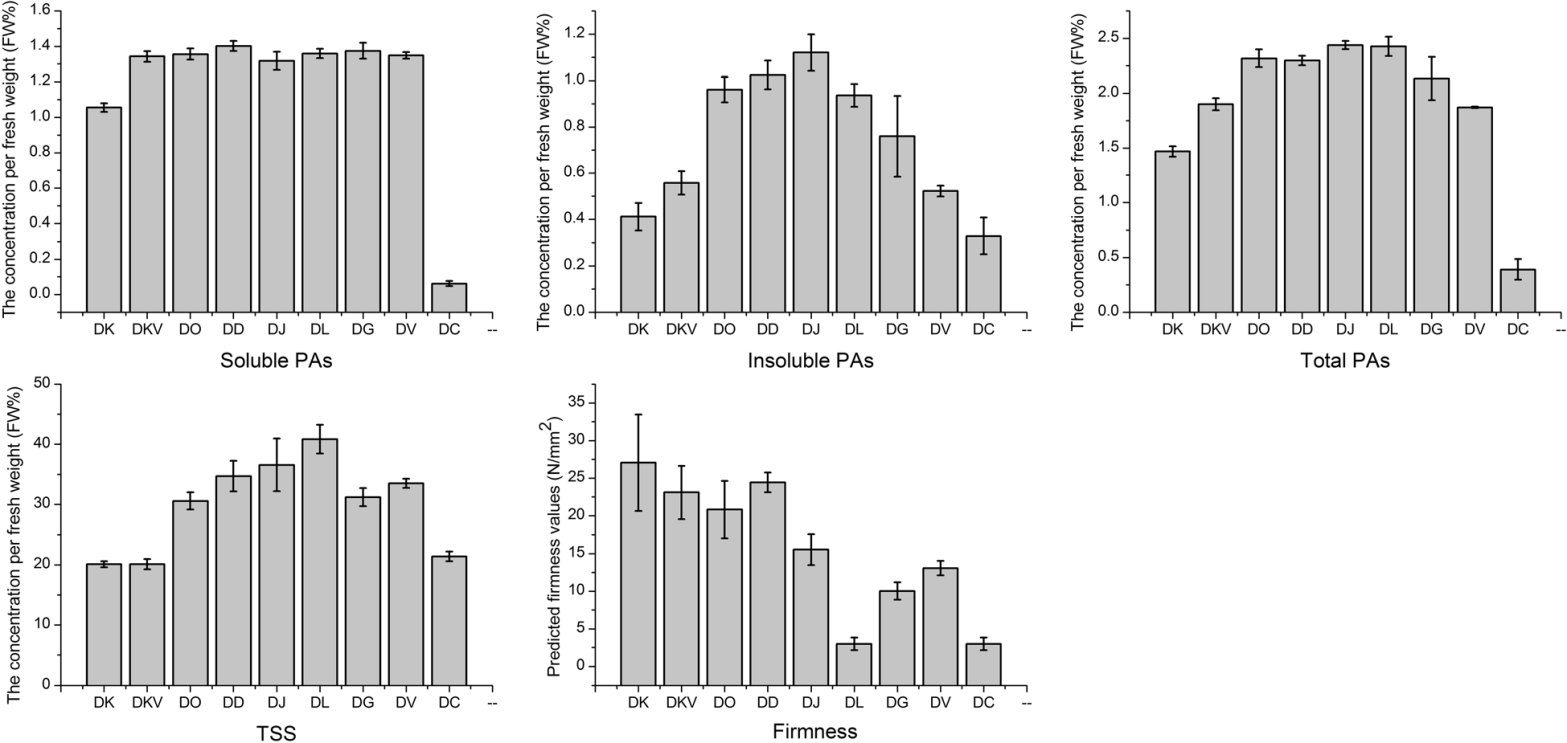
PAs, TSS and firmness of fruit in the nine *Diospyros* samples. Fruit sampled on October 31, 2018 in the NFGP. Error bars indicate standard deviation (*n* = 10)

Condensed tannins, also known as proanthocyanidins (PAs), which can be divided into soluble and insoluble PAs. Because the accumulation of soluble PAs causes astringency taste, which often is set as a reference basis for the edibility of the fruits of *Diospyros* species used as human food. The content of soluble and insoluble PAs was highest in the DD and DJ species, respectively. DC and DK species had the lowest and second-lowest level of both soluble and insoluble PAs, respectively. In general, the TSS level in softening flesh is higher than that in hard flesh for the fruit of *Diospyros*. It is noteworthy that the lowest TSS contents were observed for the DK species, this was perhaps as a result of fruit hardness effect (Fig. 2). DC and DL species were easier to soften, which have a low level of firmness at the mature stage.

### Sequencing and transcriptome assembly

The RNA-sequencing libraries were prepared from the different tissues (leaves, stems, fruit and flowers) of each *Diospyros* species or variety. Using Illumina paired-end sequencing technology, the nine samples produced approximately 400.25 million clean reads in total, averaging 44.47 million reads per sample (Additional file 5: Table S1). The de novo assembly of these high-quality reads yielded a total of 483,421 unigenes, with a mean length at 884 bp and an N50 at 1490 bp in the nine of *Diospyros* samples. Among the nine samples, the DKV species had a maximum of 90,605 unigenes, which had an average N50 length of 1225 bp and a mean length of 702 bp. The DG species had a minimum of 28,719 unigenes, which had an average N50 length of 1722 bp and a mean length of 1145 bp (Table 2).

**Table 2:** Summary of the transcriptome sequencing and assembly for the nine *Diospyros* samples

Sample	Type	Amount	GC%	N50	Min Length	Mean Length	Max Length	Assembled Bases
DJ	Genes	28689	44.9854	1713	201	1151	12091	33033702
DG	Genes	28719	45.0494	1722	201	1145	9933	32885581
DC	Genes	40712	44.7595	1540	201	934	12289	38055812
DL	Genes	45450	0.4405	1424	201	832.06	10141	37816908
DD	Genes	48012	0.438	1449	201	835.21	13906	40100032
DK	Genes	58325	0.4388	1271	201	729.06	10456	42522505
DO	Genes	67715	42.4792	1580	201	845	15802	57283383
DV	Genes	75194	42.1507	1486	201	782	15769	58859090
DKV	Genes	90605	42.008	1225	201	702	15888	63646474

### Functional annotation

The complete set of unigenes from the nine samples was annotated by homology searches using the BLASTX against the Nr, Swiss-Prot, KEGG, and COG protein databases, applying an e-value threshold of 1e-5. In total, 22,770 (79.37%) sequences from DJ, 22501(78.35%) from DG, 28649 (70.37%) from DC, 27384 (60.25%) from DL 27546 (57.37%) from DD, 30543 (52.37%) from DK, 33245 (49.10%) from DO, 35543 (47.27%) from DV, and 42,190 (46.56%) from DKV yielded at least one significant match to an existing gene model in the BLASTX searches (Table 3). In addition to the DV species, in which the two top-hits were annotated based on the genome of *Vitis vinifera*, and *Cajanus cajan*, the top species classification hits for the eight other *Diospyros* samples were *Vitis vinifera* and *Theobroma cacao* in the Nr database, which had the highest level of successful annotations (Additional file 6: Table S2).

**Table 3:** Annotation of the nine *Diospyros* sample transcriptomic libraries with Nr, Swiss-Prot, KEGG, and COG protein databases

Total Unigenes	Nr	Kegg	Swissport	Annoted	Percent (%)	Without Annotation
28689	22770	8773	17859	22788	0.794311409	5901
28719	22501	8469	17664	22529	0.784463247	6190
40712	28649	10564	21339	28726	0.705590489	11986
45450	27384	7248	19308	27467	0.604334433	17983
48012	27546	7186	19315	27586	0.574564692	20426
58325	30543	7978	21279	30784	0.527801114	27541
67715	33245	10769	23802	33454	0.494041202	34261
75194	35543	10562	24220	35671	0.474386254	39523
90605	42190	13210	28545	42391	0.467866012	48214

For the Gene Ontology (GO) functional annotation, the distribution and percentages revealed some divergence among the species, although they were similar overall (Additional file 2: Figure S1). Within the ‘biological process’ division, the cellular process and metabolic process were prominently shared in all the samples, while DD, DK and DKV had higher proportions in both the cellular process and cellular component organization/biogenesis categories. In the molecular function category, the numbers of genes annotated as transporter activity (702 unigenes) and antioxidant activity (113 unigenes) were highest in the DK species compared with those of other species, implying a possible relation to various environmental stressors. Moreover, the genes annotated as a membrane-enclosed lumen under the cellular component category were high in the DK (353 ungenes) and DD (343 ungenes) species (Additional file 2: Figure S1).

Furthermore, the COG database was used to analyze the functional prediction and phylogenetic classification of the unigenes from each species. The ‘general function prediction only’ category exhibited the largest group genes for each species, among which the DK species had the highest number of genes (9091). In the total 25 COG divisions, DK had the top proportion in the three categories, including the ‘replication, recombination’, ‘repair transcription’ and ‘coenzyme transport and metabolism’, while, for the DL species, the most represented category was ‘cell wall/membrane/envelope biogenesis’ (326 genes) (Additional file 7: Table S3).

### Ortholog identification and phylogenetic analysis

**Fig. 3:**
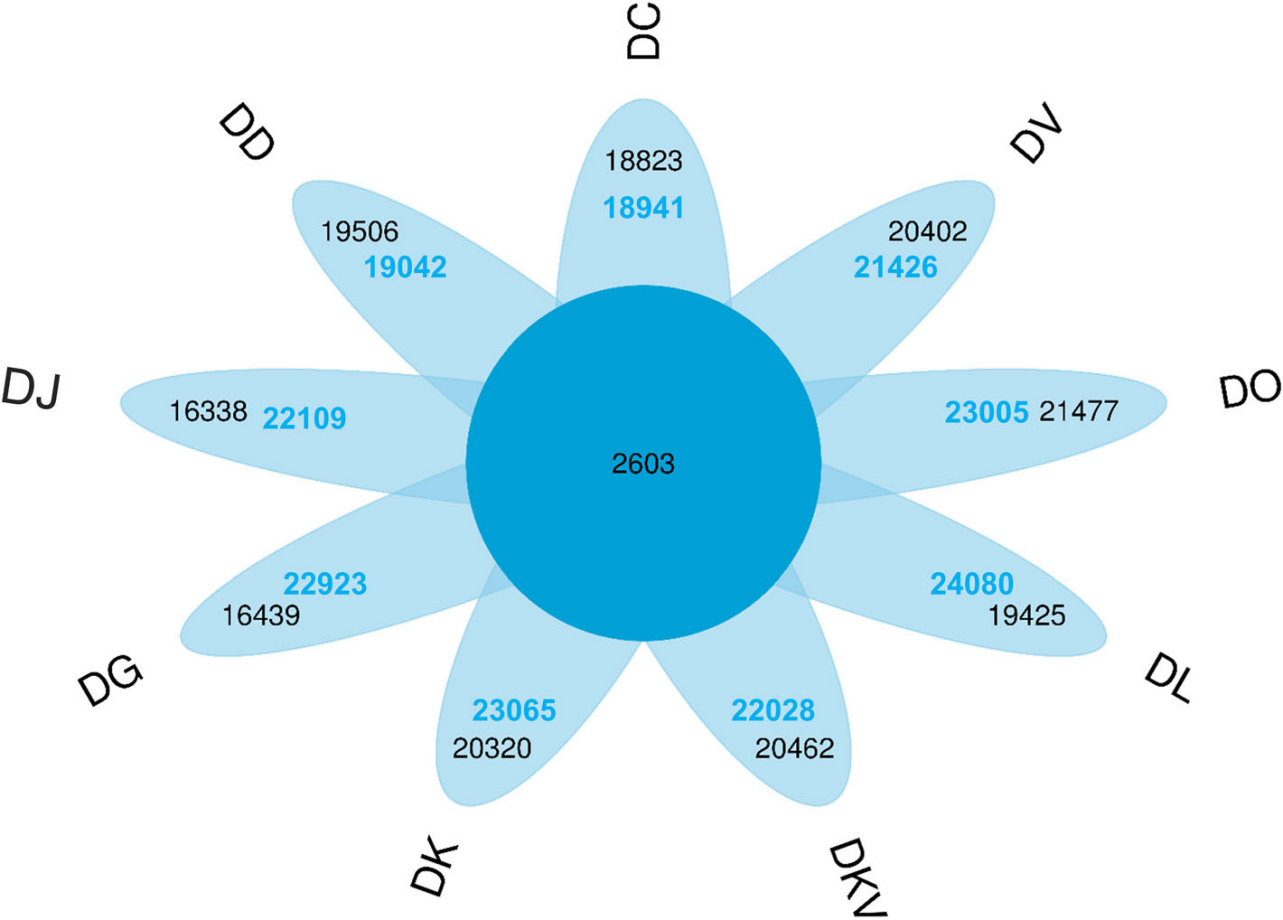
Number of PSGs shared among the species-pairs. A total of 2603 single copy orthogroups shared in the nine *Diospyros* samples. The black and blue font represent the putative orthogroups and the unigenes, respectively, in the nine *Diospyros* samples

A total of 31,242 putative orthogroups, including 196,619 unigenes, were identified based on the OrthoMCL analysis [23] (Additional file 8: Table S4). Among the 31,242 orthogroups, 2603 orthogroups were shared by all nine samples, and 22,923 orthogroups (containing 20,320 and 2603 groups) containing 58,325 genes were detected in DK (Fig. 3). DO represented a maximum of 24,080 orthogroups, and DJ showed a minimum of 18,941 orthogroups.

**Fig. 4:**
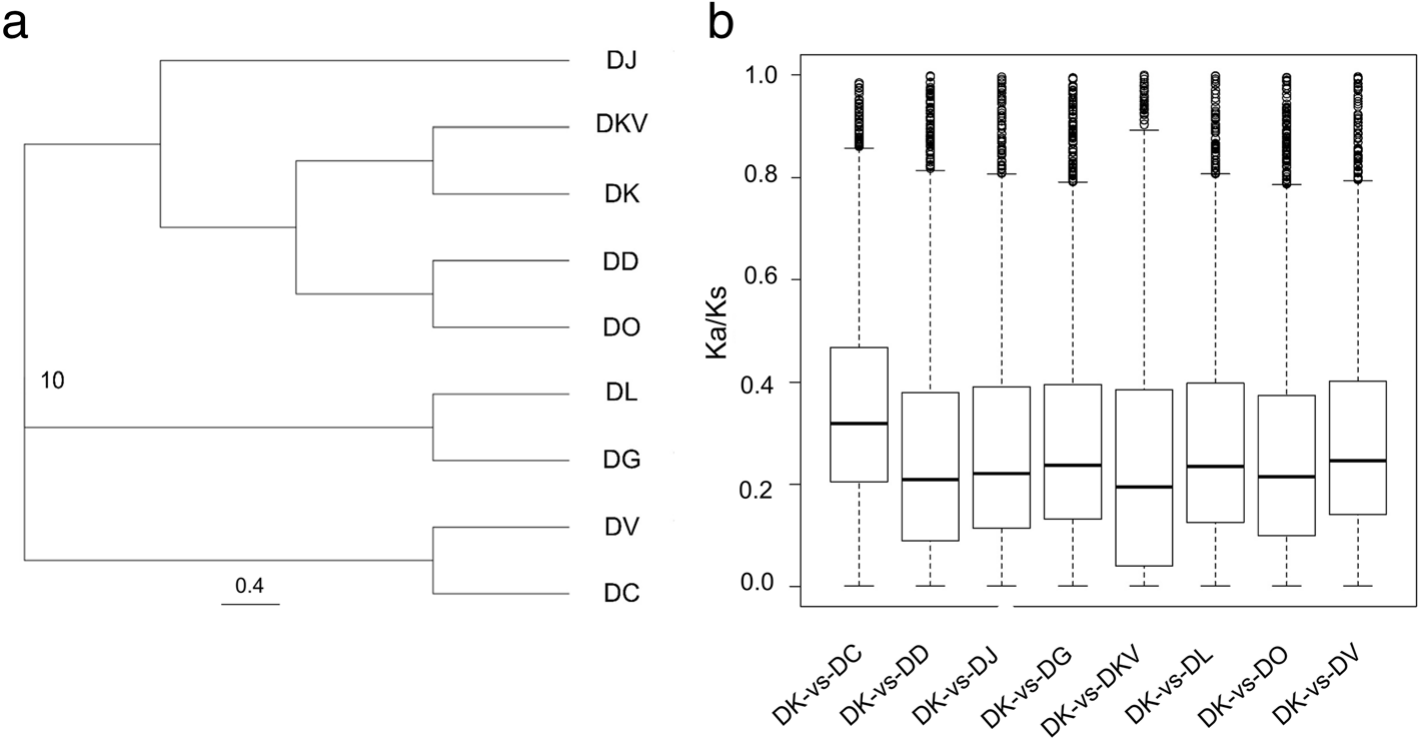
Phylogenetic relationships and nonsynonymous to synonymous mutation (Ka/Ks) ratio distributions. **a**. phylogenetic tree derived from the concatenated 2603 one-to-one single copy gene alignments (861,013 amino acid) of the nine samples, **b** Boxplots of the Ka/Ks ratios for DK compared with others species (DK-vs-DC, DK-vs-DD, DK-vs- DJ, DK-vs-DG, DK-vs-DKV, DK-vs-DL, DK-vs-DO, and DK-vs-DV)

To further confirm the phylogenetic position of persimmon, a phylogenetic tree of the nine samples was established based on 861,013 amino acid sequences (Additional file 1), which was constituted from the tandem 2603 one-to-one single copy gene alignments. The evolutionary tree consisted of three clusters (Fig. 4a). In the cluster-1 group, DK was closely related to DKV, and then it clustered with the clades of DO and DD. Cluster-3 comprised DV and DC, and the latter showed the farthest distance from the DK species. DC had the significantly smaller, narrower and evergreen leaf blades in comparison with the other taxa of *Diospyros*, which was in agreement with the morphological characteristics (Fig. 1).

### Analyses of Ka/Ks and positively selected genes (PSGs)

**Fig. 5:**
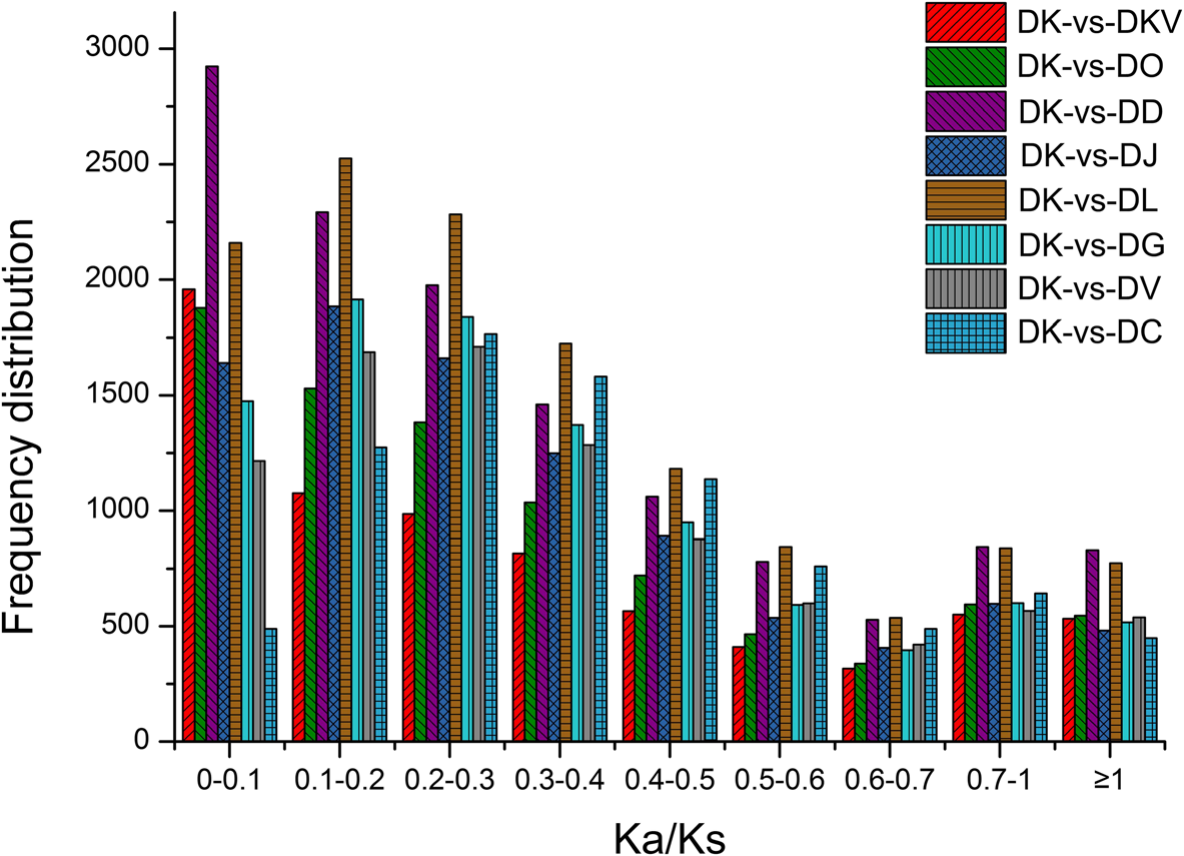
The Ka/Ks ratio distribution of the orthologs for DK compared with other species (DK-vs-DC, DK-vs-DD, DK-vs- DJ, DK-vs-DG, DK-vs-DKV, DK-vs-DL, DK-vs-DO, and DK-vs-DV)

A pairwise comparison among the 9 species was carried out with the KaKs_Calculator [24], and a total of 36 Ka/Ks ratios were obtained. Among these, 8 Ka/Ks ratios, in which DK was compared with the other species, were emphatically analyzed. The mean values of the Ka/Ks ratios from low to high were as follows: DK-vs-DJ = 0.37, DK-vs-DG = 0.37, DK-vs-DD = 0.38, DK-vs-DO = 0.38, DK-vs-DL = 0.39, DK-vs-DV = 0.40, DK-vs-DKV = 0.42, and DK-vs-DC = 0.49 (Table 4). A total of 534 and 547 ortholog pairs, from DK-vs-DKV and DK-vs-DO, had a Ka/Ks ratio ≥ 1, respectively, and both the DKV and DO species had a close relationship with DK in the cluster analysis. The highest and lowest ortholog pairs of the Ka/Ks ratio ≥ 1 were DK-vs-DD (830) and DK-vs-DC (449), respectively. The highest and lowest ortholog pairs of the Ka/Ks ratio between 0.5 and 1 were DK-vs-DL (2219) and DK-vs-DKV (1279), respectively (Table 4). A Ka/Ks ratio above 0.5 was a conservative cutoff, but it also has proven valuable for identifying genes under moderate positive selection [25]. In addition, the Ka/Ks > 1 was set as the cutoff for the positively selected genes [16]. Overall, the Ka/Ks ratios between 0 and 0.1 had the largest numbers, and the ratios between 0.6 and 0.7 had the lowest numbers (Fig. 5).

**Table 4:** List of the Ka/Ks values for all 31,242 putative orthogroups among the nine *Diospyros* samples

Comparisons	Ka/Ks		Noumbers of ortholog pairs	
	Mean value	*P*-Value (Fisher)	0.5 ≤ Ka/Ks < 1	Ka/Ks ≥ 1
DK/DJ	0.37	0.12	1835	482
DK/DG	0.37	0.12	1590	517
DK/DD	0.38	0.14	2151	830
DK/DO	0.38	0.14	1399	547
DK/DL	0.39	0.13	2219	774
DK/DV	0.40	0.12	1588	539
DK/DKV	0.42	0.16	1279	534
DK/DC	0.49	0.11	1892	449

For the 2603 single copy orthogroups shared by all nine samples that possessed both nonsynonymous and synonymous substitutions, the ratios of Ka/Ks, which were from when DK was compared with the other species, from low to high, were as follows: DK-vs-DC, DK-vs-DV, DK-vs-DG, DK-vs-DL, DK-vs- DJ, DK-vs-DO, DK-vs-DD, and DK-vs-DKV (Table 5). Although the Ka/Ks ratio of DK-vs-DO was only slightly higher than that of DK-vs-DD, their values were 0.214 and 0.209, respectively (Fig. 4b). The results of the Ka/Ks ratios from 2603 single copy sequences were consistent with the results of the previous cluster analysis. The Ka/Ks ratios were directly proportional to the genetic relationship of the different species. The higher the Ka/Ks ratios, the longer the distance was. For example, the Ka/Ks ratio of DK-vs-DC was highest, at 0.32, and the phylogenetic relationship between the DK and DC species was the farthest. In contrast, the DK and DKV species showed the closest relationship, which corresponded to the lowest Ka/Ks ratio, which was 0.20 (Fig. 4b). Moreover, a total of 16 and 39 single copy sequences, with a Ka/Ks ratio > 1 and between 0.5 and 1, were obtained from the unigene data from the DK-vs-DKV comparison, respectively (Table 5).

**Table 5:** List of the Ka/Ks values for the 2603 single copy gene shared in the nine *Diospyros* samples

Comparisons	Ka/Ks		Noumbers of ortholog pairs	
	Mean value	*P*-Value(Fisher)	0.5 ≤ Ka/Ks < 1	Ka/Ks ≥ 1
DK/DC	0.323	0.133	69	19
DK/DV	0.249	0.112	63	22
DK/DG	0.239	0.107	48	20
DK/DL	0.236	0.116	56	23
DK/DJ	0.227	0.108	49	21
DK/DO	0.214	0.107	53	17
DK/DD	0.209	0.138	41	14
DK/DKV	0.195	0.109	39	16

### Detection of candidate genes under positive selection in the DK species

To identify genes that were likely to be involved in the domestication of the cultivated species *D. kaki*, we first performed simple pairwise comparisons of the Ka/Ks ratios for obtaining the candidate PSGs; then, the rigorous criteria of PRANK (Probabilistic Alignment Kit) were applied to align the orthologs, which was considered to be more accurate and to have few false positives. Total of 152 single copy sequences (Ka/Ks > 1) were obtained from when DK was compared with the other species, and the PSGs associated with carbohydrate metabolism and phenolic metabolism showed the higher proportions. Of which, 31 PSGs associated with domestication were selected for further analysis.

The cDNA from the 31 PSGs genes was successfully isolated using homology cloning and the RACE-PCR strategy based on the RNA-seq data from the ‘Huoguan’ persimmon samples (Additional file 9: Table S5). Of these genes, 25 were full-length coding sequences (CDSs), while the others were partial CDSs. The functions of the 31 candidate PSGs were focused on carbohydrate metabolism and phenolic metabolism and mostly exhibited a potential relationship with domestication. Nearly all the 11 PSGs that were carbohydrate metabolism-related genes were focused on the metabolism of pectin, xyloglucan, and the secondary cell wall, which have a significant effect on the ripening of persimmon fruit. This dataset contained the full-length CDS of pectinesterase (PE, EC 3.1.1.11), pectin lyase (PL, EC 4.2.2.10), Beta-galactosidase (β-Gal, EC 3.2.1.23), glycosyltransferases (GT, EC 2.4.x.y), two PSGs of xyloglucan galactosyltransferase (XGT, EC 2.4.1.-), and five full-length PSGs of xyloglucan endotransglucosylase/hydrolase (XTH, EC 2.4.1.207). For the remaining 20 PSGs, almost all were involved in phenolic metabolism and contained the 18 full-length and 2 partial cDNA sequences and were associated with the reduction of soluble PAs. The astringency of persimmon fruit is usually caused by the accumulation of soluble PAs in the vacuoles. Thus, the astringency removal in the persimmon fruit is the important characteristic for cultivation and consumption. Acetaldehyde is thought to be directly involved in tannin coagulation, resulting in the loss of astringency in persimmon fruit [26], and 3 of the acetaldehyde metabolism-related genes were identified, including an alcohol dehydrogenase (ADH, EC 1.1.1.1), a pyruvate decarboxylase (PDC, EC 4.1.1.1) and a pyruvate kinase (PK, EC 2.7.1.40) gene. Moreover, 14 regulators of acetaldehyde metabolism were found, containing 5 ethylene-responsive transcription factors (ERFs) and 9 NAC transcription factors. Lastly, PA biosynthesis-related genes were obtained, such as a leucoanthocyanidin reductase (LAR, EC 1.17. 1.3) gene and 2 multidrug and toxic compound extrusion (MATE) transporter genes.

**Fig. 6:**
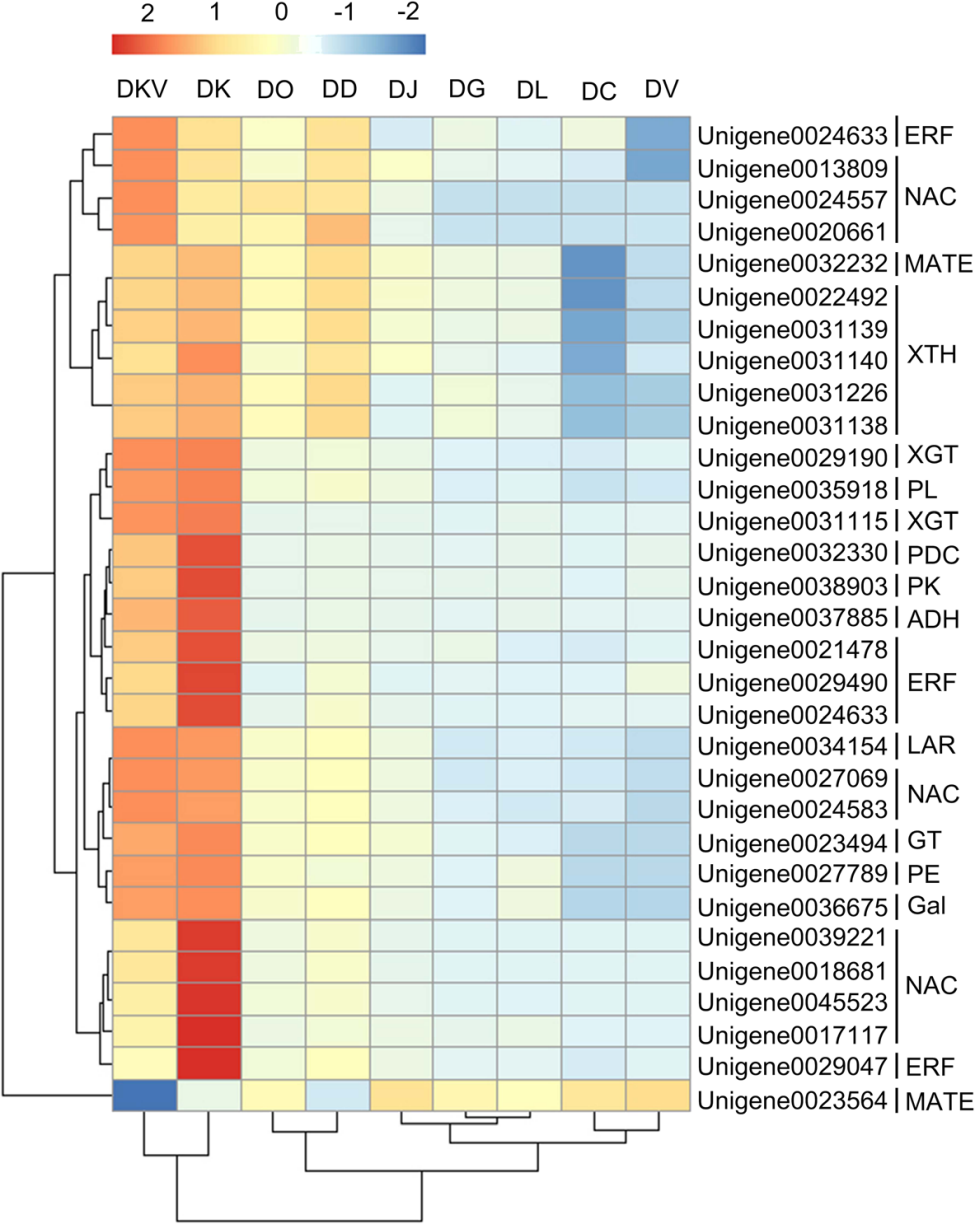
Expression pattern of candidate PSGs in the nine *Diospyros* samples. Relative mRNA expression was evaluated by qRT-PCR. The heatmap was constructed by R language (v3.0.2) indicating the average mRNA expression from 4 biological repeats. Values are colored from green (down-regulated) to red (up-regulated) according to the colour scale

To further evaluate the relationship between the 31 PSG genes and the domestication of the cultivated species DK, a qRT-PCR analysis was performed using the nine *Diospyros* samples (Additional file 9: Table S5 and Fig. 6). A total of 23 genes from 31 PSGs showed high-abundance transcript levels in the DK species. It is interesting that the expression of the *PDC*, *PK, LAR*, *ADH,* and 4 of *ERF* (Unigene0021478, Unigene0029490, Unigene0033066, and Unigene0029047), and 4 of NAC (Unigene0039221, Unigene0018681, Unigene0045523, Unigene0017117) genes were highest in the DK species, were the second-highest in the DKV species and were lowest in the DC species. They exhibited a similar pattern, overall, with the phylogenetic analysis, indicating that DK had a relationship from nearest to farthest with the DKV, DD, DO, DJ, DL, DG, DV, and DC species (Fig. 4a and Fig. 6). For the remaining 8 PSGs, the *MAET* (Unigene0023564) gene had a highest transcript level in DJ species, while others demonstrated the highest expression in DKV sample. The relatively random expression in the DK species might be due to the complex genetic characteristics of allohexaploidy. In general, most of the PSG genes had a high transcript level in the DK species, indicating that these genes were mostly related to the domestication or adaptation of the cultivated species.

In addition, the phylogenetic tree for the nine samples based on the expression of 31 PSGs was constructed (Fig. 6). It exhibited a similar pattern with the phylogenetic analysis constituted from the tandem 2603 one-to-one single copy gene alignments (Fig. 4a). DK and DKV were clustered together, and they had a close relationship with the clades of DO and DD. Whereas DK had a farthest distance from the cluster of DC and DV species (Fig. 6).

### Identification of the EST-SSRs from single copy genes in the DK species

Usually, EST-SSRs are more transferable and advantageous than random genomic SSRs for enabling reveal genetic diversity because they are likely to be more highly conserved [27]. Based on the nine *Diospyros* unigenes, a total of 53,387 SSRs were found, and the maximum and minimum numbers were from the DK (9601) and DJ (3342) species (Additional file 10: Table S6). For the 2603 single copy orthogroups shared among the nine samples, a total of 3901 SSRs were achieved from 3322 unigenes. The most abundant repeat types were trinucleotides, followed by dinucleotides (Table 6), and the most dominant repeat motifs were AG/CT, followed by AAG/CTT and CCG/CGG (Additional file 3: Figure S2). Based on the 361 SSR-containing unigenes from the 2603 single copy genes in the DK species, 432 SSR primers were identified, and 120 SSRs were selected to design the EST-SSR primers (Table 6 and Additional file 11: Table S7). All of the orthologs selected for the EST-SSR primer design had a repeat-unit length of at least 16 bp. To evaluate the reliability and transferability of these primers, we tested 50 pairs of primers for 18 individuals, representing nine samples of *Diospyros.* Of the 100 EST-SSRs, 66 SSR primers generated clear bands of the expected length, while the other PCR products exhibited more than one band, which may have resulted from the high heterozygosity. In total, 168 amplicons were detected from the 100 primer pairs. Moreover, we identified 2 unique amplicons DKSSR10 and DKSSR39: the former were absent in the DC species but were present in the other species, the latter had a long amplification product in the DJ species (Additional file 4: Figure S3). Thus, DKSSR10 and DKSSR39 could potentially serve as species-specific molecular markers to distinguish DK from other species.

**Table 6:** Summary of the microsatellite loci in the 2603 single copy orthogroups in the nine *Diospyros* samples

Data set	Unigenes containing SSRs	Total SSRs	Dinucleotide repeats	Trinucleotide repeats	Tetranucleotide repeats	Pentanucleotide repeats	Hexanucleotide repeats
DC	360	419	176	225	6	1	11
DD	375	430	168	216	9	1	14
DJ	344	404	139	244	8	2	11
DG	383	443	172	243	5	1	22
DK	361	432	169	218	7	1	12
DKV	355	420	155	230	12	3	20
DL	358	435	167	224	8	3	10
DO	413	477	192	257	11	4	13
DV	373	441	177	232	9	1	22

## Discussion

DK is the most economically important cultivated species of the genus *Diospyros* and is believed to have originated in China. However, there are few investigations about the adaptation genes and the interspecific genetic relationships in the genus *Diospyros*. In the study, nine DK samples*,* one variant and seven closely related species were sampled from the National Field Genebank for Persimmon (NFGP) in China. NFGP is currently one of the largest persimmon germplasm banks, where more than 1000 genetic resources of the genus *Diospyros*, collected from China, Japan, Korea, Israel and America, including the DK and closely related species, were preserved [28, 29]. Nevertheless, no genome sequencing data are available for the genus *Diospyros*, and only a few transcriptome sequences are reported for the DK species [30]*.* Here, the comprehensive transcriptome resource for nine *Diospyros* samples were generated and annotated to investigate their genetic relationship and detect the related-domestication genes in response to artificial selection. We generated 31,242 putative orthogroups, including 196,619 unigenes from the nine samples (Additional file 8: Table S4), and 2603 orthogroups were shared by all nine samples (Fig. 3). A phylogenetic tree of the nine samples, which constituted the tandem 2603 one-to-one single copy gene alignments (Sequence Data S1) (Fig. 4a), exhibited that the DK and DKV species clustered together and were close to the DD and DO species. These results showed a similar pattern to the previous reports, which showed that DK was closely related to DD and DO-based chloroplast genome sequences [5, 8]. It is further speculated that the hexaploid persimmon was likely to developed from the cross between the tetraploid DD and diploid DO. DK had the farthest genetic distance from DC, which was consistent with previously published results [8].

The most important quality traits for the cultivated species are their commercial value large size, high soluble sugar content and low tannin content. The genes involved in the fruit quality in persimmon were detected according to the ratio of Ka and Ks between the orthologous coding regions [14, 15]. Paired genes with Ka/Ks > 1 are determined to have experienced a rapid evolution by natural or artificial selection [16]. A Ka/Ks ratio above 0.5 is a conservative cutoff, but it has also proven valuable for identifying genes under a moderately positive selection [25]. In this study, of the 2603 single copy orthogroups shared by all nine samples, a total 152 single copy sequences had a Ka/Ks ratio > 1 when the DK was compared with other species. After manually removing some of the candidate PSGs, due to potential errors, 31 PSGs genes were successfully isolated using homology cloning and a RACE-PCR strategy based on the RNA-seq data of the ‘Huoguan’ persimmon samples (Additional file 9: Table S5). The most interesting group of genes under positive selection was related to cell wall degradation and phenolic metabolism, which was also present with the highest expression in the DK species compared to the other samples. One gene (Unigene0027789) that encoded the PE protein is involved in catalyzing the demethylesterification that influences the interactions between cell wall components, which is relevant to the softening and ripening of persimmon fruit [31]. PL degrades pectin via a β-elimination reaction that results in the formation of a 4,5-unsaturated bond at the end of the cleaved polysaccharide [32]. Two PSGs of XGT, including one full-length (Unigene0031115) and one partial CDS (Unigene0029190), are involved in the biosynthesis of xyloglucan, which is the primary cross-linking component of cellulose microfibrils [33]. GT catalyze the transfer of a sugar residue from an activated nucleotide sugar to a wide range of acceptor molecules that are associated with plant secondary metabolites, such as the biosynthesis of the secondary cell wall and the production of phenolic volatiles [34].

In this study, the genes *PDC*, *PK, ERF*, and *LAR*, which are related to phenolic metabolism, had the highest expression in the DK species, which had a lower PA content in persimmon fruits than in the other species except DC sample in general (Fig. 2). This finding is consistent with the previous reports that the high expression of the genes *PDC*, *PK, ERF*, and *LAR* promote the deastringency of persimmon fruits [35, 36]. It should be noted that these genes exhibited a similar pattern (Fig. 6), overall, with the phylogenetic analysis (Fig. 4a), indicating that DK had a relationship, from nearest to farthest, with the DKV, DD, DO, DJ, DL, DG, DV, and DC species. Moreover, other genes related to fruit ripening or softening are also under positive selection, including genes encoding XTH (Unigene0031138, Unigene0031226, Unigene0031140, Unigene0022492, Unigene0031139), and *β-Gal* gene. XTH is thought to play a major role in fruit ripening via loosening the cell wall and accelerating fruit softening [37]. β-Gal is involved in cell wall biogenesis and modification during fruit softening by cleaving beta galactan bonds [38]. Their high expression might lead to earlier ripening in persimmon than in other species. However, there were x genes present at a low expression in the DK species, which might be due to the complex genetic characteristics of allohexaploidy and needs further study in the future. In general, most PSG genes had the high transcript level in the DK species, which further provides evidence that these genes were mostly related to the domestication or adaptation of the cultivated species.

The development of the SSRs in the genus *Diospyros* was mostly focused on the DK species using the traditional enriched genomic library [39]. Recently, it was reported that 42,711 SSR loci were found using Roche 454 sequencing technology, and 98 revealed polymorphisms between 15 DK genotypes [40]. However, some closely related species with DK still lack the SSR information. In this study, we developed the EST-SSR based on the RNA-Seq data in nine *Diospyros* samples, including a variety of persimmon and seven wild species. A total of 53,387 SSRs were obtained from the 41,586 unigenes among the nine samples. Those novel SSR markers from the *Diospyros* species were more useful for genetic studies and breeding applications. Tri-nucleotide repeats were the most abundant repeat motifs, followed by di-, and tetra-nucleotide repeats, which was consistent with previous reports [41].

Moreover, 100 SSRs were selected to design the EST-SSR primers (Table 6 and Additional file 11: Table S7) from 2603 single copy orthogroups shared in nine samples, and 66% products exhibited more than one band in all nine samples. Our PCR success rate was higher than that in a previous study [40], but it was slightly lower than those reported in rubber [42]. The failure of 34 primer pairs to produce amplicons may be due to the location of the primers across the splice sites, chimeric primers, poor-quality sequences, or the high heterozygosity. Most products presented only one band, which might result from the homozygosity of the loci in *Diospyros* samples, for which all the primers were from the single copy genes. In addition, our study found that DKSSR10 and DKSSR39 were unique in the DC and DJ species respectively (Additional file 7: Figure S3), thus they could potentially serve as species-specific molecular markers to distinguish the *Diospyros* species.

## Conclusion

In this study, we performed transcriptome sequencing on nine samples, including DK, a variety of DK (DKV) and seven closely related species (DJ, DC, DV, DO, DG, DD, and DL), to evaluate the interspecific genetic divergence and to identify candidate genes involved in persimmon domestication. A total of 483,421 unigenes were assembled by 400.25 million clean reads, and 2603 orthogroups shared among all the samples were obtained. A phylogenetic tree was established based on the tandem 2603 one-to-one single copy gene alignments, showing that DK was closely related to DKV, clustered with the clade of DD and DO species. These findings were further supported that the persimmon was developed from the cross between the DD and DO species. Furthermore, 31 PSGs, which are involved in carbohydrate metabolism and phenolic metabolism, were identified and isolated, and all had a higher expression in the DK or DKV species. It was determined that those genes might contribute to the domestication of the DK species. Finally, we identified 2 unique amplicons (DKSSR10 and DKSSR39) that could potentially serve as species-specific molecular markers to distinguish the *Diospyros* species. Our analysis suggests candidate PSGs that may be crucial for the adaptation, domestication, and speciation of persimmon relatives.

## Materials and methods

### Materials

Nine samples containing eight *Diospyros* species, namely, DJ, DC, DV, DO, DG, DD, DL, DK, and DKV (a variety of DK, also known as wild persimmon) were obtained from NFGP, Yangling, Shaanxi, China (34°17′42.80″N, 108°04′8.21″E) (Fig. 1 and Table 1). Different tissues, including the leaves, stems, fruit and flowers, were sampled from at least 10 individuals of each species. All the samples were quick-frozen with liquid nitrogen and were stored at − 85 °C. The samples for physiology evaluation and morphological characteristics were collected on October 31, 2018 in the NFGP.

### Fruit physiology evaluation

Soluble and insoluble Proanthocyanidins (PAs) were measured according to our previous report, using the Folin–Ciocalteu method [31]. The absorbance was measured at 725 nm using a UV-2450 spectrophotometer (Shimadzu, Japan). Soluble solids content (SSC) was measured using a digital refractometer of Atago PR-101 (PR-101, Atago, Co., Japan). Firmness were determined by GY-4 hardness tester (Top instrument CO., LTD., China) according to the instruction manual. A cylindrical probe 11.1 mm in diameter was pressed in the peeled fruit to a depth of 10 mm. Penetration tests were carried out at a rate of 5 mm/s (2 s to maximum depth). The SSC and firmness was measured on each of 10 fruit per treatment at the fruit equator.

### RNA extraction and Illumina sequencing

The total RNA from each sample was isolated using the RNAplant Plus Reagent (Tiangen Biotech Co., Beijing, China) according to the manufacturer’s protocols. The RNA quality and quantity were assessed using a NanoDrop 2000 spectrophotometer (Thermo Fisher Scientific, Waltham, MA, USA) and RNase-free agarose gel electrophoresis. Equal amounts of high-quality mRNA from the different tissues (leaf, stems, fruit and flowers) of each species were pooled for cDNA library construction and Illumina sequencing. For sequencing, the cDNA libraries were generated according to the manufacturer’s protocol using the NEBNext Ultra™ RNA Library Prep Kit for Illumina Inc. (NEB, USA). Then, the library preparations were sequenced using Illumina HiSeq PE150 platform (Illumina, San Diego, CA, USA). Library construction and Illumina sequencing were performed at Sagene Biotech Co., Ltd. (Guangzhou, China).

### De novo assembly and functional annotation

The raw data generated from the Illumina sequencing were filtered by removing the adapter sequences, the reads that were less than 20 bp, the reads with a proportion of unknown base calls (Ns) that was higher than 5%, and the reads with a percentage of low-quality bases (Q < 5) of more than 50%. The transcriptome de novo assembly from each library was carried out with the Trinity program [43].

The assembled unigenes from the final transcriptome were annotated by homology searches using the Basic Local Alignment Search Tool (BLASTX) against the Nr (NCBI nonredundant protein database, http://www.ncbi.nlm.nih.gov/), Swiss-Prot (A manually annotated and reviewed protein sequence database, http://www.expasy.ch/sprot/), KEGG (Kyoto Encyclopedia of Genes and Genomes, http://www.genome.jp/kegg/), and COG (Cluster of Orthologous Groups of proteins, http://www.ncbi.nlm.nih.gov/COG/) protein databases, applying an e-value threshold of 1e-5. The directions of the unigene sequences were determined based on the high scoring alignments or the best alignment results. Incongruent alignment results, which were generated from the different databases, were settled following a priority order of Nr, Swiss-Prot, KEGG, and COG. When a unigene did not match entries in any of the databases, the ESTScan program [44] was used to identify the coding regions and sequence directionality.

The Gene Ontology (GO) terms of the unigenes were obtained using the Blast2GO [45], and the GO functional classifications were calculated by the Web Gene Ontology Annotation Plot (WEGO) software [46] to view the distribution of the gene functions of the species at the macro level. The COG classification and KEGG metabolic pathway annotations were performed using Blastall software against the COG [47] and KEGG [48] databases, respectively. The enrichment analysis used custom Perl scripts.

### Ortholog identification and phylogenetic analysis

The identification of the orthologs among the 9 *Diospyros* samples was performed using the OrthoMCL analysis. The OrthoMCL software [23] was selected to calculate the clusters of the orthologs with an e-value <1e-15 and an inflation parameter of 2.0. Unigenes with lengths less than 150 bp were excluded. Only orthologous gene clusters with at least one sequence per species were used in the further analyses for Ka/Ks and PSGs. The putative orthologous groups with only single copy genes (one to one orhologs) that were shared by all samples were then aligned using MUSCLE software [49] with the default parameters. The single copy gene was that the only one gene per samples in the ortholgous groups. A phylogenetic tree (Unrooted PHYLIP tree) for putative orthologous groups with only single copy genes was then constructed in the program ClustalX using the Neighbor-Joining method [50] (1000 bootstraps).

### Analyses of Ka/Ks and PSGs

The ratio of Ka and Ks for each ortholog pair was estimated between the orthologous coding regions in KaKs_Calculator [24] using the maximum-likelihood method with the YN model. In this study, the synonymous and nonsynonymous sites, Ka/Ks ratios, GC contents, and the sequence length removing stop codons and gaps for each ortholog pair were calculated [51]. A series of rigorous criteria were used to ensure the reliability of PSGs [51–55]. Firstly, the orthologous sequence pairs with *P*-value (Fisher test) > 0.05 or a Ks rate > 0.1, were excluded from the identification of the PSGs to avoid potential paralogs [52, 53]. Secondly, the ortholog sequences were retained for the further analysis when they showed a best hits with each other by MegaBLAST method and their aligned length exceeded 150 bp [54, 55]. Thirdly, aligned sequences with unexpected stop-codons were excluded from further analysis [51]. Lastly, Ka/Ks > 1 was set as the cutoff for the PSGs showing that the genes have experienced a rapid evolution by natural or artificial selection [16]. A Ka/Ks ratio > 0.5 is suggested as an appropriate threshold value for genes under moderate positive selection [25].

### Quantitative reverse transcription PCR (qRT-PCR)

qRT-PCR was carried out in the ABI One Step Plus Real-Time PCR System (Applied Biosystems, CA, USA). The PCR reaction mixture (20 μl total volume) included 10 μl of SYBR® Premix Ex Taq™ II (Tli RNaseH Plus) (TaKaRa, Dalian, China), 7.4 μl of ddH_2_O, 1.0 μl of diluted cDNA, and 0.8 μl of each primer (10 μM). The standard PCR conditions, with three steps, were as follows: 4 min at 95 °C; then 45 cycles of 95 °C for 5 s; 57 °C for 30 s; and 72 °C for 30 s. *DkActin* [56] was set as the internal reference, each sample was performed in quadruplicate, and all the primers are listed in Additional file 9: Table S5.

## Additional files

Additional file 1: Alignments of 861,013 amino acid sequences from the tandem 2603 one-to-one single copy gene. (FASTA 2522 kb)

Additional file 2: **Figure S1.** Gene ontology (GO) classifications of the assembled nonredundant unigenes in the nine *Diospyros* samples. The results are summarized in A: ‘biological process’, B: ‘cellular component’, and C: ‘molecular function’. The x- and y-axes represent the GO categories and the total number of all the unigenes, respectively. (JPG 555 kb)

Additional file 3: **Figure S2.** The total distribution frequency of the SSR repeat motifs from the 2603 orthogroups shared in *Diospyros* Linn. (JPG 382 kb)

Additional file 4: **Figure S3.** Polymorphism and validation of a subset of the microsatellite primer pairs for nine *Diospyros* samples by agarose-gel profiling. 1–16 represent DD, DD, DK, DK, DC, DC, DG, DG, DO, DO, DKV, DKV, DJ, DJ, DV, DV, DL, DL, respectively. M: marker. (JPG 190 kb)

Additional file 5: **Table S1.** Databases for the de novo sequencing of the transcriptome for the nine *Diospyros* samples. (XLSX 10 kb)

Additional file 6: **Table S2.** Ten top-hits for the nine *Diospyros* samples annotated using the NCBI NR database with BLASTX. (XLSX 11 kb)

Additional file 7: **Table S3.** COG classification for the nine *Diospyros* samples. (XLSX 99 kb)

Additional file 8: **Table S4.** Putative orthogroups among the nine *Diospyros* samples identified based on the OrthoMCL analysis. (XLSX 3354 kb)

Additional file 9: **Table S5.** Primer sequences and Nr-annotation for 31 PSGs. (XLSX 12 kb)

Additional file 10: **Table S6.** Summary of the EST-SSRs identified from the orthogroups in the nine *Diospyros* samples. (XLSX 10 kb)

Additional file 11: **Table S7.** Characteristics of the 120 conserved microsatellites primers based on orthologs in the DK species. (XLSX 21 kb)

## Abbreviations

ADH: Alcohol dehydrogenase
CDSs: Coding sequences
DC: *Diospyros cathayensis*
DD: *Diospyros deyangnsis*
DG: *Diospyros glaucifolia*
DJ: *Diospyros jinzaoshi*
DK: *Diospyros kaki*
DKV: *Diospyros kaki* var. silvestris (a variety of DK, also known as wild persimmon)
DL: *Diospyros lotus*
DO: *Diospyros* oleifera
DV: *Diospyros virginiana*
EST-SSR: Expressed sequence tag-simple sequence repeat
GO: Gene Ontology
GT: Glycosyltransferases
IRAPs: Inter-retrotransposon amplified polymorphisms
ITS: Internal transcribed sequence
Ka: Nonsynonymous substitutions
Ks: Synonymous substitutions
LAR: Leucoanthocyanidin reductase
MATE: Multidrug and toxic compound extrusion
NGS: Next-generation sequencing
PAs: Proanthocyanidins
PDC: Pyruvate decarboxylase
PE: Pectinesterase
PK: Pyruvate kinase
PL: Pectin lyase
PSGs: Positively selected genes
REMAPs: Retrotransposon microsatellite amplified polymorphisms
XGT: Xyloglucan galactosyltransferase
XTH: Xyloglucan endotransglucosylase/hydrolase
β-Gal: Beta-galactosidase
