# Supplementary figures and images for: Comparative transcriptomic analysis reveals genetic divergence and domestication genes in Diospyros
Source: BMC Plant Biol. 2019 May 30;19:227. doi: 10.1186/s12870-019-1839-2 (PMC6543618; doi:10.1186/s12870-019-1839-2)

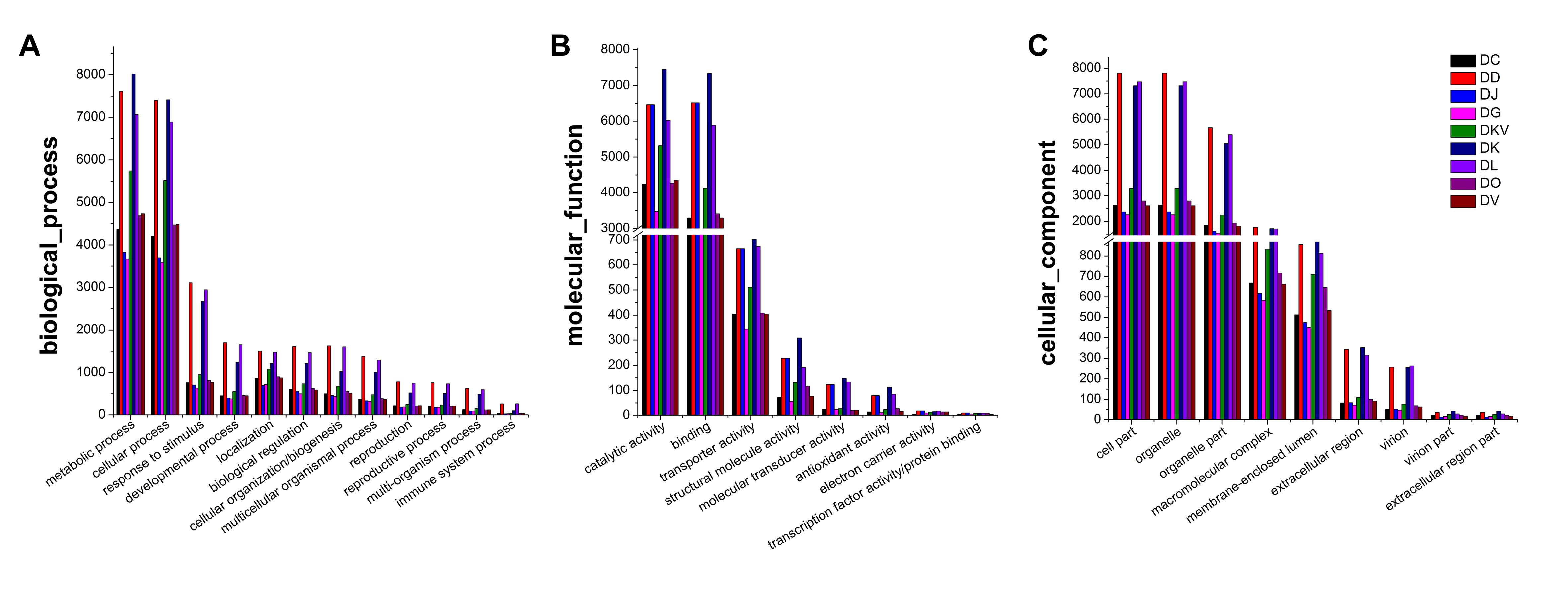

Supplement: Supplementary file 2 — Figure S1. Gene ontology (GO) classifications of the assembled nonredundant unigenes in the nine Diospyros samples. The results are summarized in A: ‘biological process’, B: ‘cellular component’, and C: ‘molecular function’. The x- and y-axes represent the GO categories and the total number of all the unigenes, respectively. (JPG 555 kb) [file 12870_2019_1839_MOESM2_ESM.jpg]

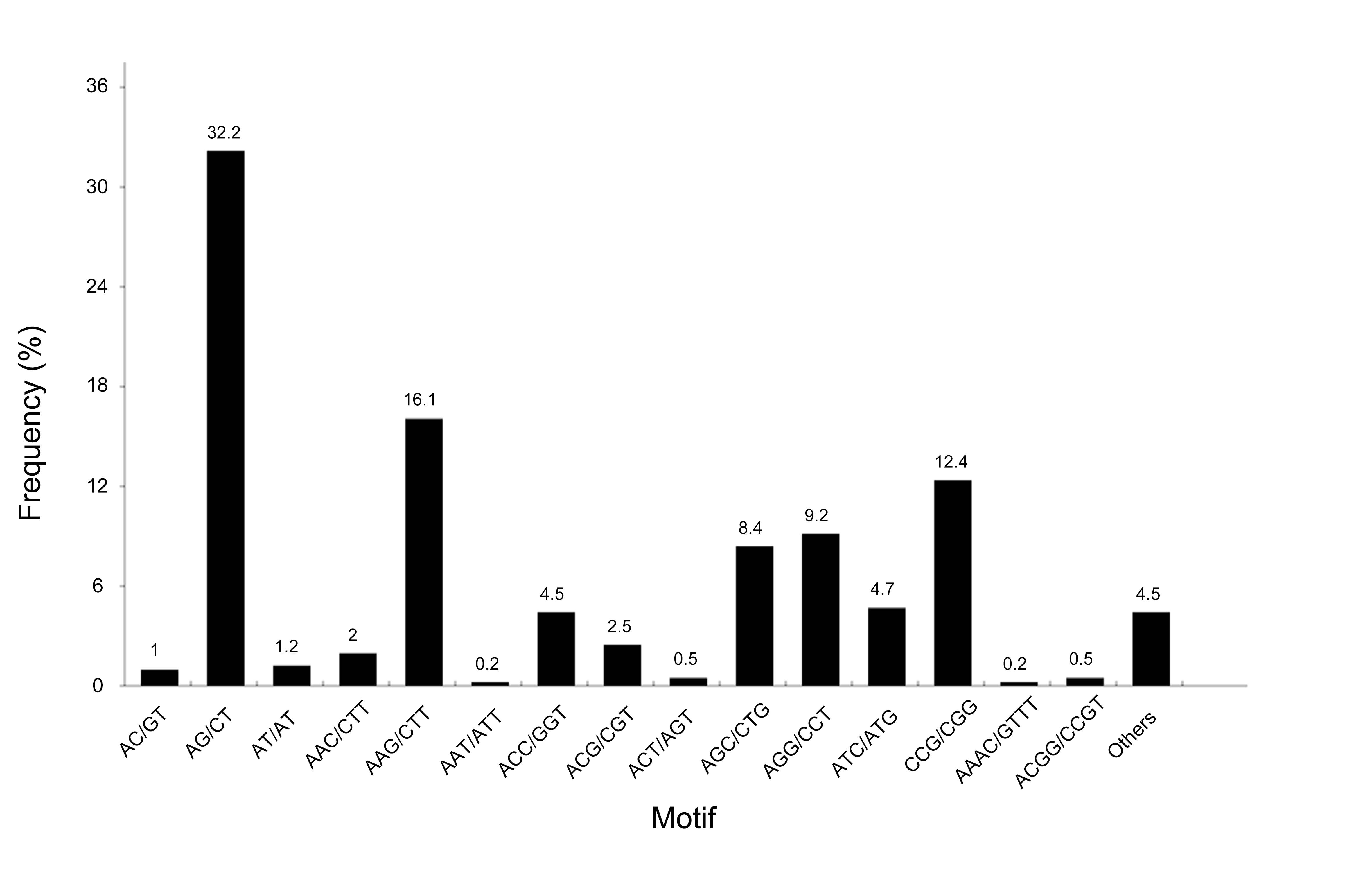

Supplement: Supplementary file 3 — Figure S2. The total distribution frequency of the SSR repeat motifs from the 2603 orthogroups shared in Diospyros Linn. (JPG 382 kb) [file 12870_2019_1839_MOESM3_ESM.jpg]

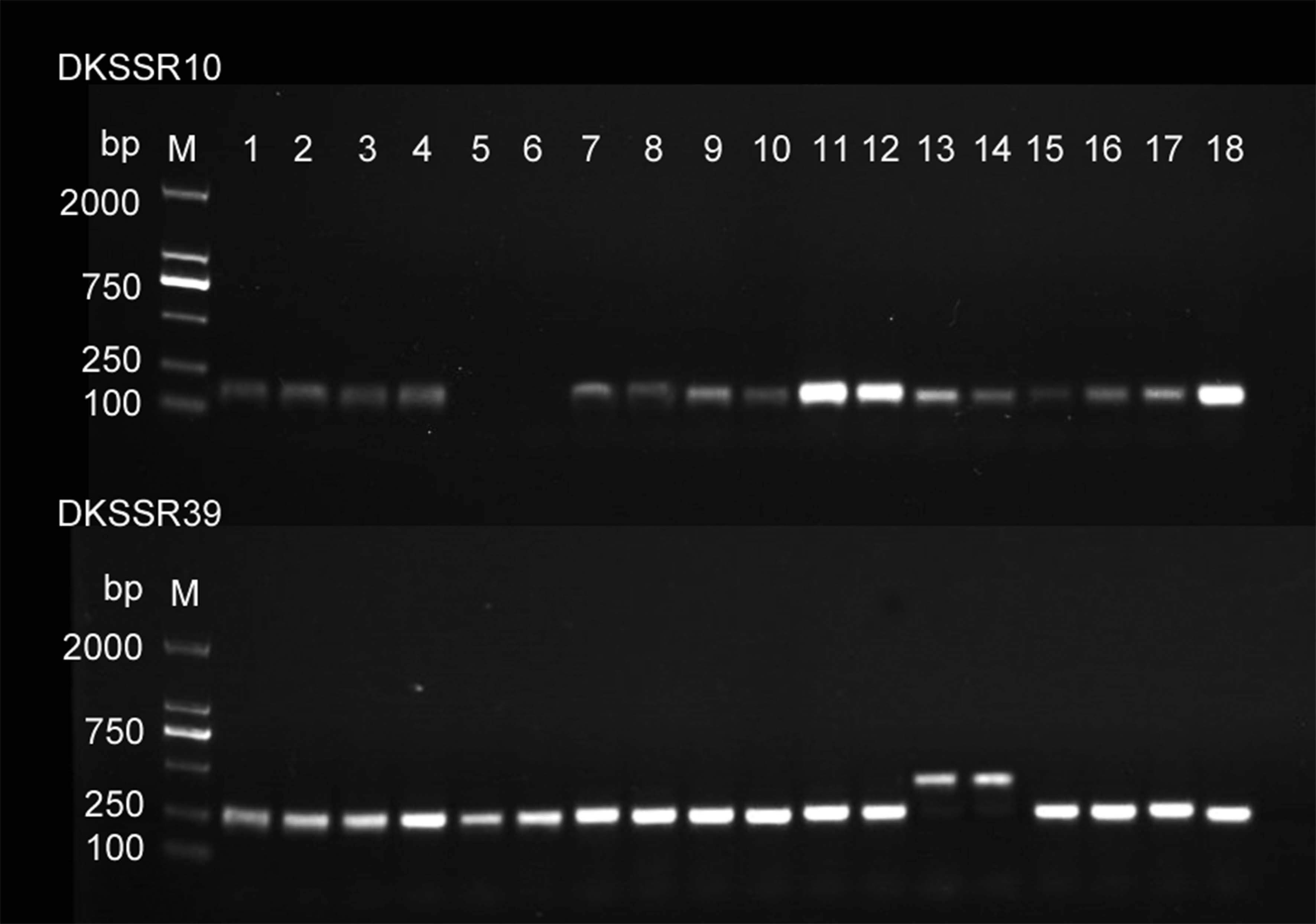

Supplement: Supplementary file 4 — Figure S3. Polymorphism and validation of a subset of the microsatellite primer pairs for nine Diospyros samples by agarose-gel profiling. 1–16 represent DD, DD, DK, DK, DC, DC, DG, DG, DO, DO, DKV, DKV, DJ, DJ, DV, DV, DL, DL, respectively. M: marker. (JPG 190 kb) [file 12870_2019_1839_MOESM4_ESM.jpg]
